# Supplementary material for: X-ray structure of a carpet-like antimicrobial defensin–phospholipid membrane disruption complex
Source: Nat Commun. 2018 May 17;9:1962. doi: 10.1038/s41467-018-04434-y (PMC5958116; doi:10.1038/s41467-018-04434-y)
Supplement: Supplementary file 1 — Supplementary Information [file 41467_2018_4434_MOESM1_ESM.pdf]

Supplementary Information

X-ray structure of a carpet-like antimicrobial defensin–phospholipid membrane disruption complex

Michael Järvå[1], Fung T. Lay[1], Thanh Kha Phan[1], Cassandra Humble[1], Ivan K. H. Poon[1], Mark R. Bleackley[1], Marilyn A. Anderson[1], Mark D. Hulett[1]*, Marc Kvansakul[1]*

[1]Department of Biochemistry and Genetics, La Trobe Institute for Molecular Science, La Trobe University, Melbourne, Victoria, 3086, Australia.

* Correspondence should be addressed to M.K. (email: M.Kvansakul@latrobe.edu.au) or M.D.H. (email: M.Hulett@latrobe.edu.au)

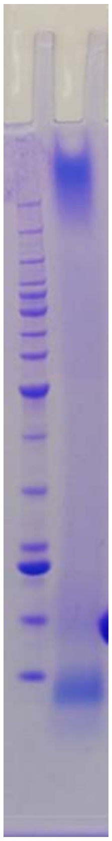

Supplementary Figure 1. Non-reducing SDS-PAGE analysis of NaD1:PA complex. Non-reducing SDS-PAGE analysis of NaD1:PA complex revealing the formation of a large molecular weight complex.

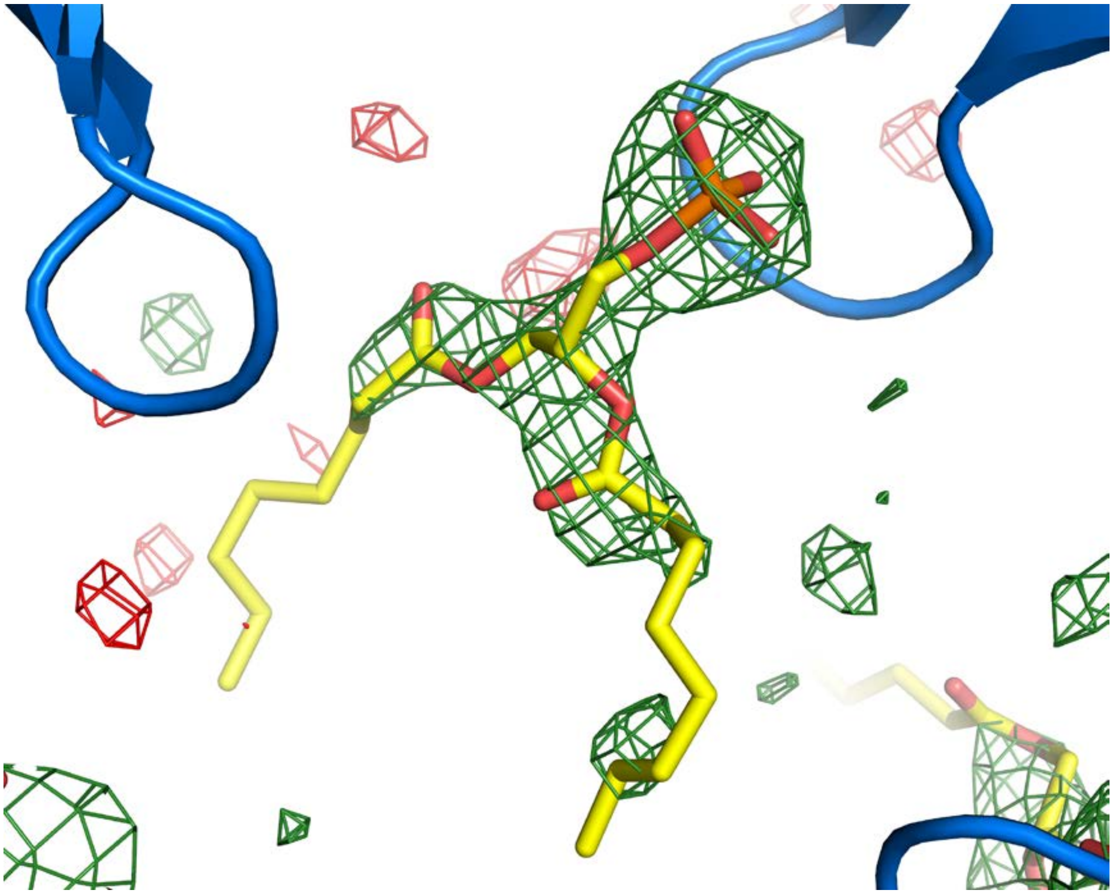

Supplementary Figure 2. PA binding site in NaD1-PA oligomer. Representative PA binding site with NaD1 in cartoon representation (blue) and the PA molecule as sticks (carbon yellow, oxygen red, and phosphoros orange). FoFc density maps were generated by removing the PA molecules prior to the final refinement. Maps are contoured at 3.0 sigma in PyMol and overlaid with the final structure.

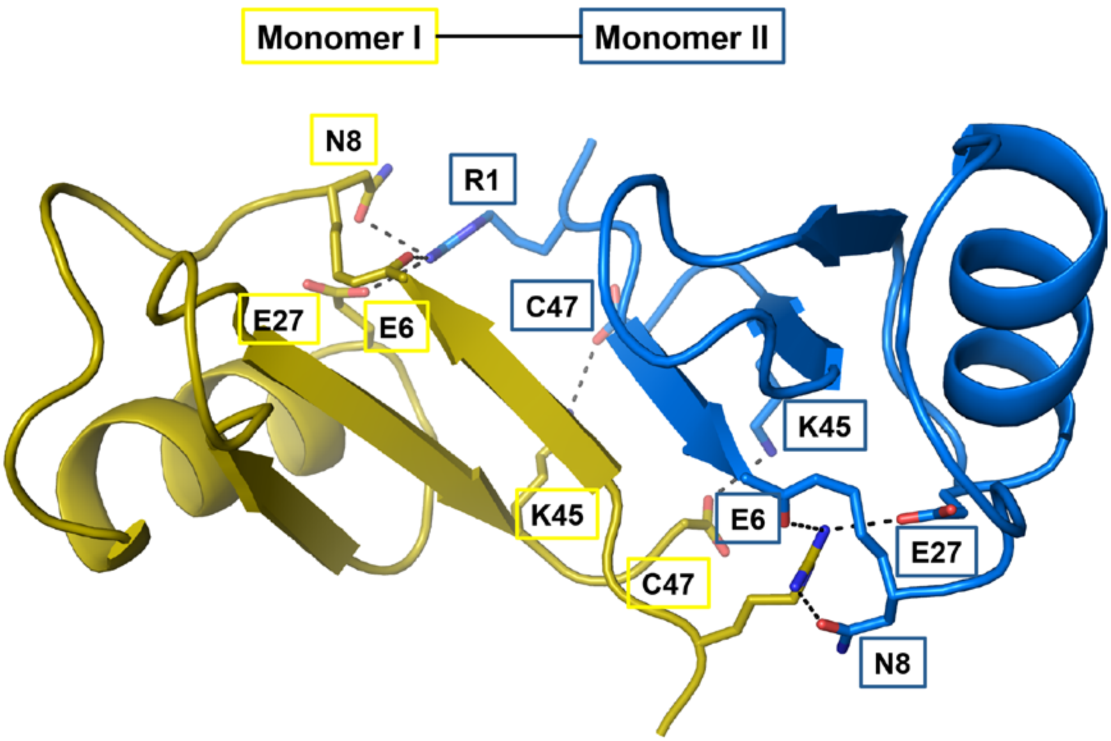

Supplementary Figure 3. NaD1 dimer formation. One NaD1 dimer in cartoon representation (yellow and blue), with residues involved in quarternary interactions depicted as sticks (colored by atom type). The NaD1 dimers are held together through β-sheet formation between β1 and β1 (not shown), and through Arg1−Glu6/Asn8/Glu27, and Lys45−Cys47.

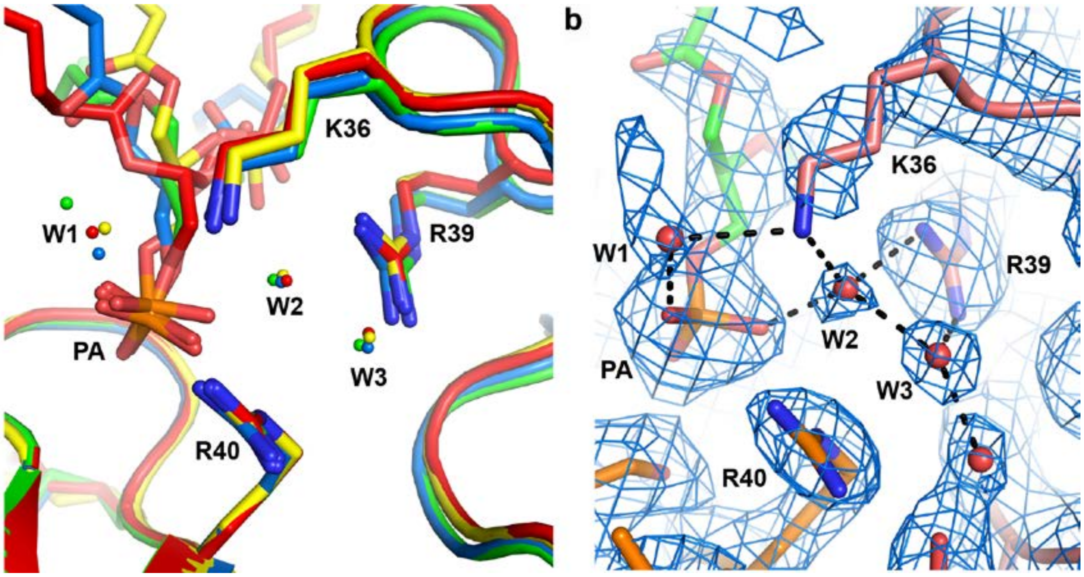

Supplementary Figure 4. A water network in the PA binding site. The PA binding site employs cooperative binding of the PA molecules by recruiting additional dimers via three conserved waters, Lys36, and Arg39. **(a)** Overlay of the four binding sites of the middle arc (cartoon representation, with the side chains of Lys36, Arg39, and Arg40 as sticks, PA molecules as sticks, and water molecules as spheres) which shows the identical positioning of the cooperative binding residues Arg39 and Lys36 and the three waters (W1-W3) that create the hydrogen bond network with PA. These waters are conserved throughout all 14 PA binding sites (not shown here for clarity). **(b)** A representative view of one binding site with the 2FoFc electron density map depicted in blue mesh contoured at the 2.0 sigma level. Hydrogen bonds involving the three water molecules are depicted as dashed black lines.

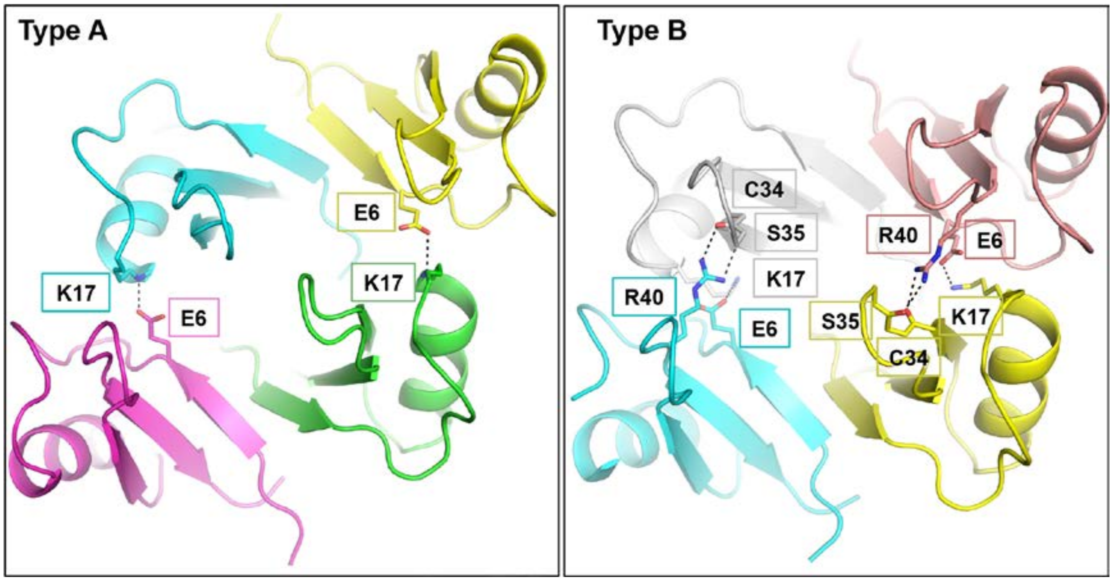

Supplementary Figure 5. NaD1 dimer interfaces. The membrane disruption complex there has seven dimer–dimer interfaces. Six of the type A configuration (left) and one of the type B configuration (right). The type A configuration comprises only two hydrogen bonds between the pairs of Glu6 and the backbone of Lys17. The type B configuration, which is only present in the middle pair of the middle arc of the membrane disruption complex, has the same Gly6–Lys17 hydrogen bond as in type A, but has an additional Arg40 interaction with the backbones of Cys34 and Ser35.

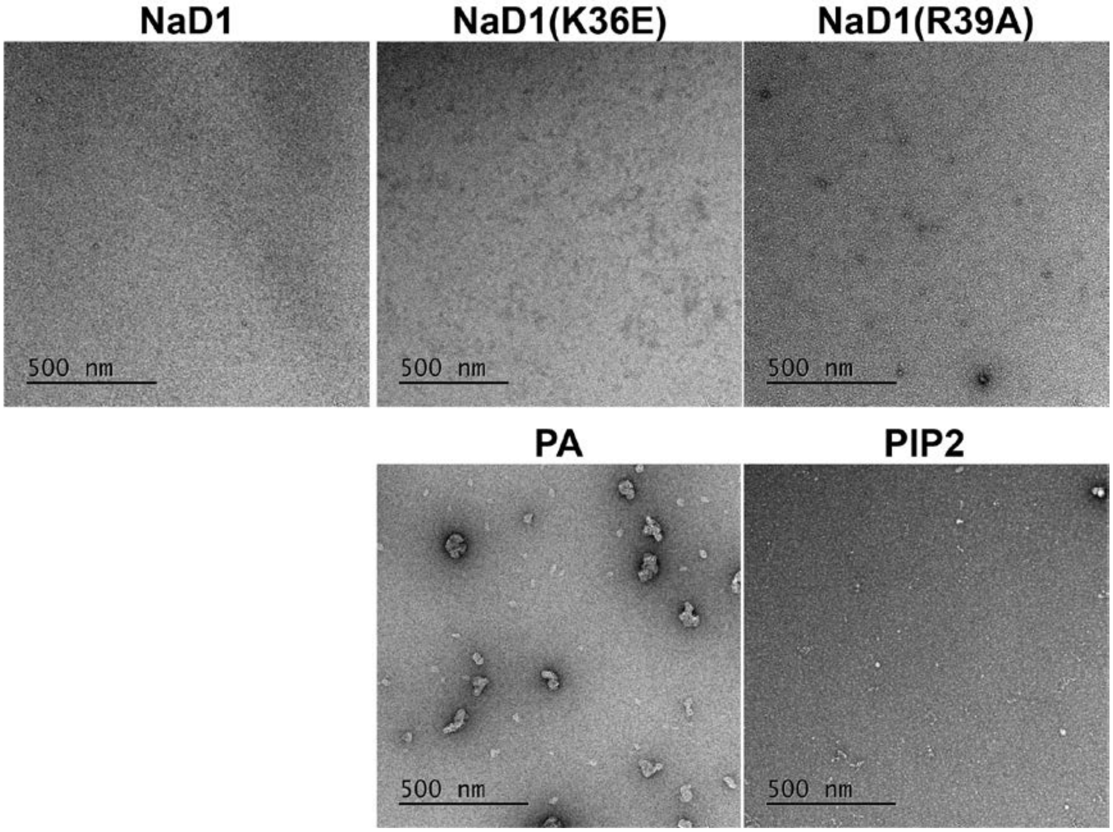

Supplementary Figure 6. Negative stain TEM analysis. Transmission electron micrographs of NaD1, NaD1(K36E), and NaD1(R39A) without added lipids (top row), and of PA and PIP_2 alone (bottom row).

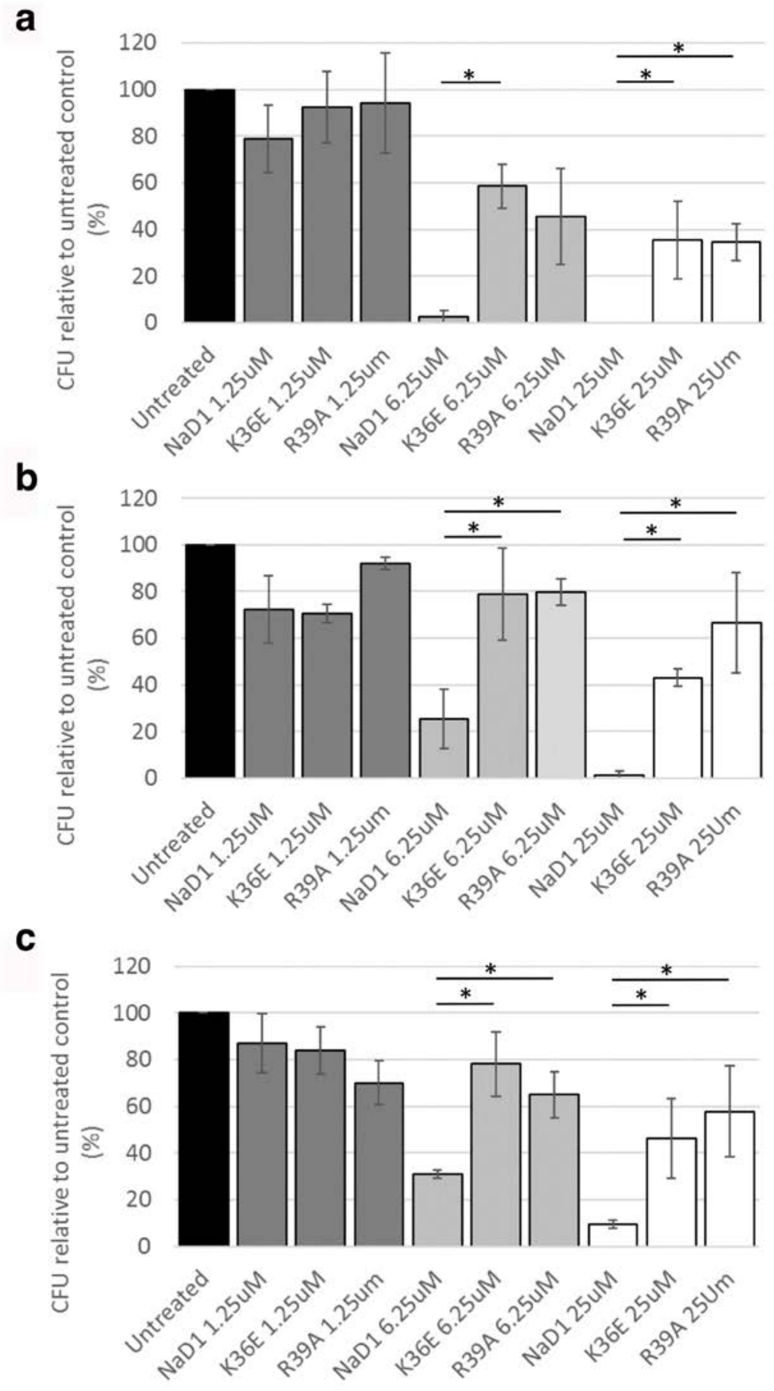

Supplementary Figure 7. *C. albicans* cell survival after treatment with NaD1 variants. *C. albicans* cells were treated in ½ PDB with two NaD1 variants, K36E and R39A, as well as the wild type defensin for 30 min, diluted 1:1000 in PBS and then plated on YPD agar. Cell survival was calculated as the number of colony forming units after 24 h incubation compared to the untreated control. The assay was performed with 3 *C. albicans* strains **(a)** LTUMC001, **(b)** ATCC10231, **(c)** ATCC90028. All values are the average of 3 independent biological replicates, error bars are SEM. * $p<0.05$ (one-tailed t-test).

Supplementary Table 1

Primer sequences	
**Construct**	Wild-type NaD1
**Forward primer (5' → 3')**	CTC GAG AAA AGA GCT AGA GAA TG
**Reverse primer (5' → 3')**	GCG AAT TAA TTC GCG GCC GC
