# Supplementary material for: X-ray structure of a carpet-like antimicrobial defensin–phospholipid membrane disruption complex
Source: Nat Commun. 2018 May 17;9:1962. doi: 10.1038/s41467-018-04434-y (PMC5958116; doi:10.1038/s41467-018-04434-y)
Supplement: Supplementary file 3 — Description of Additional Supplementary Files [file 41467_2018_4434_MOESM3_ESM.pdf]

Description of Additional Supplementary Files

File Name: Supplementary Movie 1

Description: NaD1-PA oligomer assembly. This movie demonstrates the different parts that constitute the full membrane disruption complex and how it packs within the crystal. Each dimer, in cartoon view, is shown in yellow and blue, and each phosphatidic acid molecule is shown as space filling spheres with carbon in green, oxygen in red, and phosphorous in orange.
